# The human cuneate nucleus contains discrete subregions whose neurochemical features match those of the relay nuclei for nociceptive information

**DOI:** 10.1007/s00429-013-0625-4

**Published:** 2013-08-23

**Authors:** Marina Del Fiacco, Marina Quartu, Maria Pina Serra, Marianna Boi, Roberto Demontis, Laura Poddighe, Cristina Picci, Tiziana Melis

**Affiliations:** 1 Department of Biomedical Sciences, Section of Cytomorphology, University of Cagliari, Cittadella Universitaria, 09042 Monserrato, Italy; 2 Department of Public Health, Clinical and Molecular Medicine, Azienda Ospedaliero Universitaria, University of Cagliari, 09042 Monserrato, Italy

**Keywords:** Nucleus cuneatus, Dorsal column nuclei, Immunohistochemistry, Human medulla oblongata, Somatosensory neurotransmission, Pain

## Abstract

The present paper is aimed at defining distinctive subdivisions of the human cuneate nucleus (Cu), evident from prenatal to old life, whose occurrence has never been clearly formalized in the human brain, or described in other species so far. It extends our early observations on the presence of gray matter areas that host strong substance P (SP) immunoreactivity in the territory of the human Cu and adjacent cuneate fascicle. Here we provide a three-dimensional reconstruction of the Cu fields rich in SP and further identify those areas by means of their immunoreactivity to the neuropeptides SP, calcitonin gene-related peptide, methionine- and leucine-enkephalin, peptide histidine-isoleucine, somatostatin and galanin, to the trophins glial cell line-derived neurotrophic factor and brain-derived neurotrophic factor, and to the neuroplasticity proteins polysialylated neural cell adhesion molecule and growth-associated protein-43. The presence, density and distribution of immunoreactivity for each of these molecules closely resemble those occurring in the superficial layers of the caudal spinal trigeminal nucleus (Sp5C). Myelin and Nissl stainings suggest that those Cu subregions and the Sp5C superficial layers share a similar histological aspect. This work establishes the existence of definite subregions, localized within the Cu territory, that bear the neurochemical and histological features of sensory nuclei committed to the neurotransmission of protopathic stimuli, including pain. These findings appear of particular interest when considering that functional, preclinical and clinical studies show that the dorsal column nuclei, classical relay station of fine somatic tactile and proprioceptive sensory stimuli, are also involved in pain neurotransmission.

## Introduction

The dorsal column-medial lemniscus system has classically been viewed as a pathway involved in conveying somatic epicritic tactile and kinesthetic sensory information to the thalamus. Several synaptic neurotransmitters have been identified in the dorsal column nuclei. Glutamate (Galindo et al. 1967; Roberts 1974; De Biasi and Rustioni 1990; De Biasi et al. 1994) and glycine and GABA (Galindo et al.^1967^; Roberts 1974; Rustioni et al. 1984; Westman 1989b; Heino and Westman 1991; Popratiloff et al. 1996; Lue et al. 2001) are, respectively, considered the primary excitatory and inhibitory neurotransmitters, whereas ATP (Galindo et al. 1967), acetylcholine (Henderson and Sherriff 1991), monoamines (Blomqvist and Broman 1993; Maqbool et al. 1993), and various neuropeptides (Vincent et al. 1985; Taber-Pierce et al. 1985; Westman 1989a; Tamatani et al. 1989; Conti et al. 1990; Fabri and Conti 1990) may also intervene as transmitters and/or modulators. As a general rule, the chemical neuroanatomy of second-order nuclei that relay protopathic sensibility, including nociceptive input, namely the spinal dorsal horn, the caudal part of the spinal trigeminal nucleus (Sp5C) and the solitary nucleus, differs from that of the dorsal column nuclei. This is particularly evident for a number of neuropeptides. Thus, for instance, compared to dorsal column nuclei, protopathic sensory nuclei host greater amounts of nerve fibers and terminals (and, for some neuropeptides, perikarya) immunoreactive to substance P (SP) (Cuello and Kanazawa 1978; Ljungdahl et al. 1978; Del Fiacco et al. 1984), calcitonin gene-related peptide (CGRP) (Skofitsch and Jacobowitz 1985b; Unger and Lange 1991; van Rossum et al. 1997), methionine- (M-EK) and leucine-enkephalin (L-EK) (Simantov et al. 1977; Haber and Elde 1982; Conrath-Verrier et al. 1983), somatostatin (SOM) (Johansson et al. 1984; Taber-Pierce et al. 1985; Vincent et al. 1985; Chigr et al. 1989), and galanin (GAL) (Skofitsch and Jacobowitz 1985a; Melander et al. 1986; Kordower et al. 1992). Second-order sensory nuclei for protopathic and epicritic sensation differ in a similar way in their content of other neuroactive substances, such as the trophins brain-derived neurotrophic factor (BDNF) (Tang et al. 2010; Yan et al. 1997) and glial cell-derived neurotrophic factor (GDNF) (Jongen et al. 1999; Quartu et al. 1999; Kawamoto et al. 2000; Del Fiacco et al. 2002), and markers of neuronal plasticity, such as the highly polysialylated neural cell adhesion molecule (PSA-NCAM) (Bonfanti et al. 1992; Seki and Arai 1993; Quartu et al. 2008) and the growth-associated protein-43 (GAP-43) (Benowitz et al. 1988; Wiese et al. 1991; Nacimiento et al.^1993^; Del Fiacco et al. 1994; Jain et al. 1995; Quartu et al. 1995; Zou and Martin 1995). On the other hand, early studies in our laboratory (Del Fiacco et al. 1983, 1984) showed that the human cuneate nucleus (Cu) and adjacent fascicle contain discrete gray matter subregions that host a very rich plexus of nerve fibers and terminals strongly immunoreactive to SP, a neuropeptide that, while showing a neuroregulatory action on a variety of functions, appears definitely involved in pain transmission (Hökfelt et al. 1977; Pearson et al. 1982; De Koninck et al. 1992). This peculiar localization was detectable in specimens from subjects at age ranging from prenatal life to old age (Del Fiacco et al. 1983, 1984) and has been subsequently confirmed in the human infant (Rikard-Bell et al. 1990). It also appeared as a distinctive feature of the human medulla oblongata, which has never been reported in other species, so far. Our later studies showed that immunoreactivity to other molecules, namely GAP-43 (Quartu et al. 1995), GDNF family ligands (Del Fiacco et al. 2002; Quartu et al. 2007b), and their cognate receptors (Quartu et al. 2007a), also labels distinctively some restricted subregions within the territory of the Cu and cuneate fascicle. Here we present further data showing the position and extent of the strongly SP-immunoreactive fields within the Cu territory of the human newborn medulla oblongata and provide evidence that, in newborn and adult tissue, the same discrete gray matter subregions host immunoreactivity to several other neuropeptides, neurotrophins GDNF and BDNF, and neuroplasticity proteins PSA-NCAM and GAP-43, with distribution pattern and density that strictly match those of the Sp5C; finally, the localization of those subregions in the adult medulla oblongata is illustrated by reference to the relevant diagrams of the human brainstem nuclei from Paxinos et al. (2012). The main goal of the present paper is to definitely determine the existence of such peculiar subdivisions of the human Cu, constantly detectable from developmental age to old life, and add information regarding their position and neurochemical properties.

## Materials and methods

Specimens of medulla oblongata were obtained at autopsy from subjects with no signs of neuropathology, at age ranging 21 gestation weeks to 88 years (Table 1). The sampling and handling of human specimens conformed to the local Ethics Committee of the National Health System in compliance with the principles enunciated in the Declaration of Helsinki. Fixation in 4 % freshly prepared phosphate-buffered formaldehyde, pH 7.3, for 4–6 h at 4 °C, was followed by overnight rinsing in 0.1 M phosphate buffer (PB), pH 7.3, containing 5–20 % sucrose. Transverse sections of the medulla oblongata were cut with a cryostat at 10–14 or 30 μm and collected in series of adjacent slices on chrome alum-gelatin coated slides. The indirect immunofluorescence (Coons and Kaplan 1950) in single and double staining, and the avidin–biotin-peroxidase complex (ABC) immunostaining techniques were applied using primary antibodies against neuropeptides SP, CGRP, M-EK, L-EK, peptide histidine-isoleucine (PHI), SOM and GAL, trophins GDNF and BDNF, and neuroplasticity marker molecules GAP-43 and PSA-NCAM. Details on primary and secondary antibodies and staining protocols are listed in Table 2. In ABC stainings, the immunoreaction was revealed with 30 min incubation in ABC (BioSpa Div.), diluted 1:250, followed by incubation with a solution of 0.1 M PB, pH 7.3, containing 0.05 % 3-3′-diaminobenzidine (Sigma), 0.04 % nickel ammonium sulfate, and 0.01 % hydrogen peroxide. All antisera and ABC were diluted in phosphate-buffered saline (PBS) containing 0.2 % Triton X-100. Double-labeling immunofluorescence for SP and a second peptide was attained with the rat monoclonal antibody against SP and a second primary antibody raised in rabbit either by incubating the sections in a mixture of the primary antibodies followed by a mixture of the appropriate secondary antisera, or by performing sequentially two single immunoreactions for SP and one of the other neuropeptides. Negative control preparations were obtained either by incubating tissue sections with diluted primary antibody that had been preabsorbed with 10 mM of the respective peptide for 24 h at 4 °C, with preabsorption of the anti-PSA-NCAM antibody with 5 mg of colominic acid (alfa-2-8-linked sialic polymer colominic acid), or by omitting the primary antibody. In double immunolabeling, cross preabsorption of each primary antibody with the other peptide did not alter the staining pattern. Cresyl violet, Black-Gold II staining kit (Biosensis) and Kluver-Barrera method were used as Nissl and myelin stainings. Slides stained by immunofluorescence were mounted in glycerol/PBS 3:1 (V/V); slides stained by ABC immunoperoxidase, cresyl violet, Black-Gold II kit and/or Kluver-Barrera technique were dehydrated and coverslipped. Observations and photographs were made with epifluorescence equipped photomicroscopes Leitz Dialux 20 and Olympus BX61, and with Nanozoomer 2.0-RS (Hamamatsu). Using the photographs of a series of 37 consecutive sections of medulla oblongata dorsal quadrant, the three-dimensional rendering of the SP-immunoreactive areas present in the Cu of a full-term newborn was obtained by the software Rhinoceros 5.0 (Robert McNeel & Associates). Diagrams of appropriate levels of the human medulla oblongata nuclei from Paxinos et al. (2012), reduced to the dorsal quadrant and simplified by removing lettering of non-pertinent structures, have been paired to CGRP-immunostained sections of adult medulla oblongata to show the localization of the identified subregions in the adult.

**Table 1:** List of specimens

Case	Age	Sex	Primary cause of death	Post-mortem (h)
1	Fetus 21 w.g.	F	Cardiorespiratory failure	29
2	Fetus 38 w.g.	M	Gestosis	38
3	Pre-term newborn 6 d (25 w.g.)	F	Pneumonitis	25
4	Pre-term newborn 4 d (34 w.g.)	M	Pneumonitis	32
5	Pre-term newborn 1 d (34 w.g.)	M	Cardiorespiratory failure	29
6	Pre-term newborn (38 w.g.)	M	Cardiorespiratory failure	38
7	Full-term newborn (40 w.g.)	M	Cardiorespiratory failure	28
8	Full-term newborn 17 h	F	Patency of Botallo’s duct	26
9	Full-term newborn 1 d	M	Cardiorespiratory failure	24
10	Full-term newborn 2 d	F	Persistence of fetal circulation	38
11	Full-term newborn 55 h	M	Cardiorespiratory failure	30
12	Full-term newborn 7 d	F	Cardiorespiratory failure	27
13	Adult 32 y	M	Hypertrophic cardiomyopathy	24
14	Adult 42 y	M	Cardiorespiratory failure	49
15	Adult 44 y	M	Stabbing	40
16	Adult 46 y	M	Pneumonitis	31
17	Adult 48 y	F	Myocardial infarction	24
18	Adult 53 y	F	Cardiorespiratory failure	31
19	Adult 53 y	F	Pneumonitis	28
20	Adult 56 y	F	Cardiomyopathy	34
21	Adult 71 y	M	Renal failure	25
22	Adult 72 y	F	Acute pulmonary edema	27
23	Adult 77 y	F	Myocardial infarction	26
24	Adult 88 y	M	Thromboembolysm of pulmonary artery	47

*F* female, *d* days, *h* hours, *M* male, *y* years, *w.g.* weeks of gestation (calculated from the 1st day of the latest menstrual cycle)

**Table 2:** Primary and secondary antibodies used

	Source	Dilution	Incubation
Primary antibody			
Rat monoclonal anti-SP	Cuello et al. (1979); Sera Lab, NCL 52 20064	1:300	1 h at room temperature or overnight at 4 °C
Rabbit polyclonal anti-SP	CRB, CA-08-335	1:1,200	Overnight at 4 °C
Rabbit polyclonal anti-CGRP	Peninsula Labs, RAS-6009-N	1:300	1 h at room temperature or overnight at 4 °C
Rabbit polyclonal anti-M-EK	Probert et al. (1983)	1:300	Overnight at 4 °C
Rabbit polyclonal anti-L-EK	Wharton et al. (1980)	1:300	Overnight at 4 °C
Rabbit polyclonal anti-PHI	Bishop et al. (1984); CRB, CA-08-310	1:200	Overnight at 4 °C
Rabbit polyclonal anti-SOM	Incstar Corp., 20088	1:200	Overnight at 4 °C
Rabbit polyclonal anti-GAL	CRB, CA-08-250	1:1,000	Overnight at 4 °C
Rabbit polyclonal anti-GDNF	SantaCruz Biotechnology	1:400	Overnight at 4 °C
Rabbit polyclonal anti-BDNF	SantaCruz Biotechnology	1:400	Overnight at 4 °C
Mouse monoclonal anti-PSA-NCAM	Chemicon	1:400	Overnight at 4 °C
Mouse monoclonal anti-GAP-43	Schreyer and Skene (1991)	1:1,000	Overnight at 4 °C
Secondary antibody			
DTAF-conjugated goat anti-rat serum	Jackson	1:50	40–60 min at room temperature
TRITC-conjugated goat anti-rat serum	Jackson	1:50	40–60 min at room temperature
FITC-conjugated sheep anti-rabbit serum	Wellcome	1:40	40–60 min at room temperature
DTAF-conjugated goat anti-rabbit serum	Jackson	1:100	40–60 min at room temperature
TRITC-conjugated goat anti- rabbit serum	Jackson	1:100	40–60 min at room temperature
Biotin-conjugated goat anti-mouse serum	Jackson	1:50	60 min at room temperature
Biotin-conjugated goat anti-rabbit serum	Jackson	1:100	60 min at room temperature
Biotin-conjugated goat anti-rabbit serum	Vector	1:200	60 min at room temperature

## Results

**Fig. 1:**
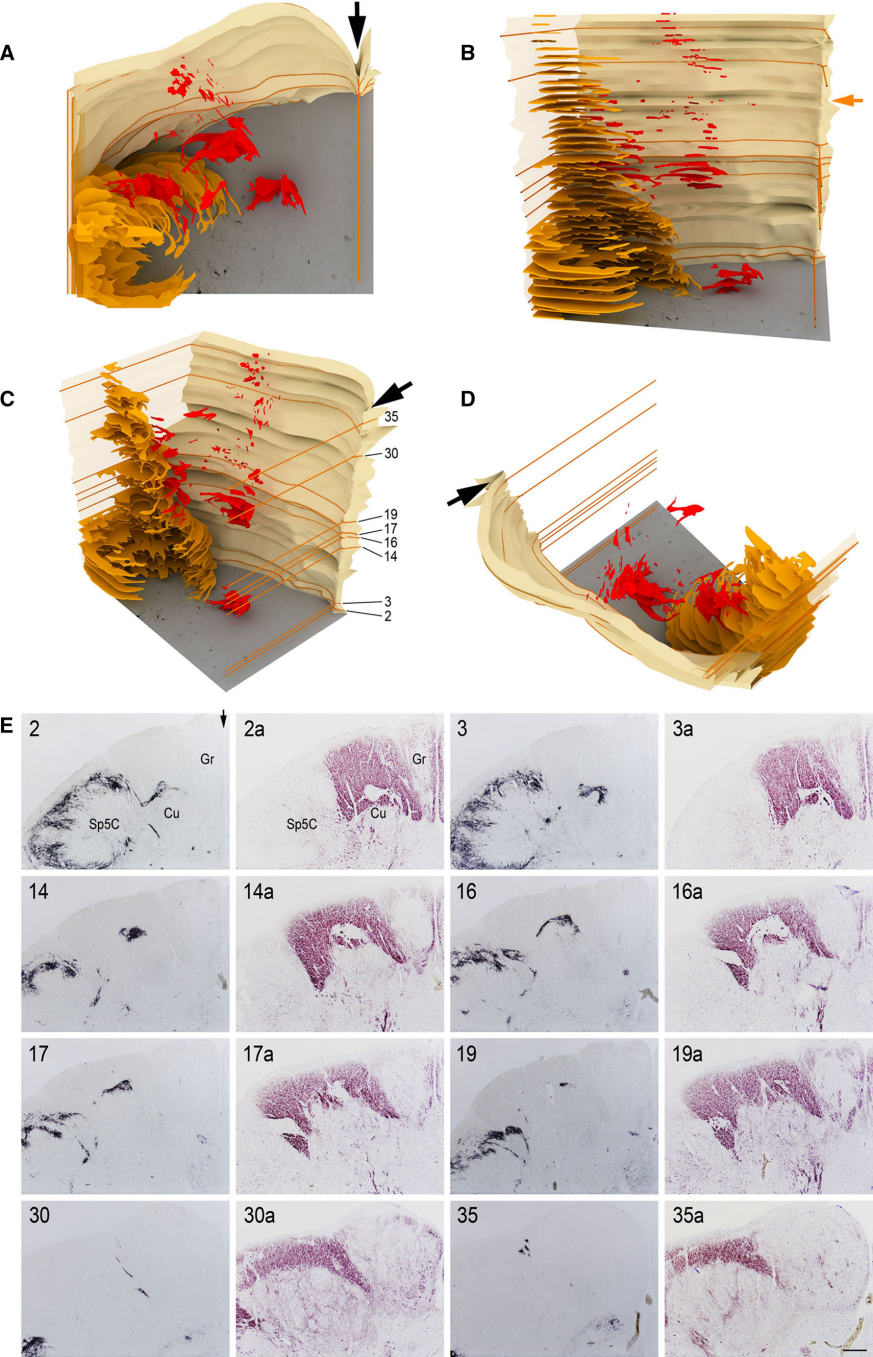
Full-term newborn, case 9. **A**–**D** 3D rendering of SP-immunoreactive areas in the territory of the cuneate nucleus and fascicle (*red*) and in the substantia gelatinosa of the spinal trigeminal nucleus, caudal part (*yellow*) as seen in thirty-seven 12 μm thick serial sections of the medulla oblongata left dorsal quadrant. Sections are distant 120 μm from one another, for a total thickness of about 4.5 mm; the top level is about 740 μm caudal to the obex. *Arrows* in **A**, **C**, **D** and **E** *2* point to the posterior median sulcus and septum; the midsagittal plane was used to align the section images. Views from the top (**A**), anterior (**B**), anteromedial-superior (**C**), dorsolateral-superior (**D**). Levels of sections *2*, *3*, *14*, *16*, *17*, *19*, *30*, and *35* in caudo-rostral sequence are indicated by orange lines and are numbered in **C**. Corresponding sections and paired, 36 μm distant, sections stained for myelin are shown in **E**. *Orange arrow* in **B** indicates the level of the caudal pole of the external cuneate nucleus. **E** *Cu* cuneate nucleus, *Gr* gracile nucleus, *Sp5C* spinal trigeminal nucleus, caudal part. *Scale bar* in **E** *35a* = 500 μm applies to all micrographs

**Fig. 2:**
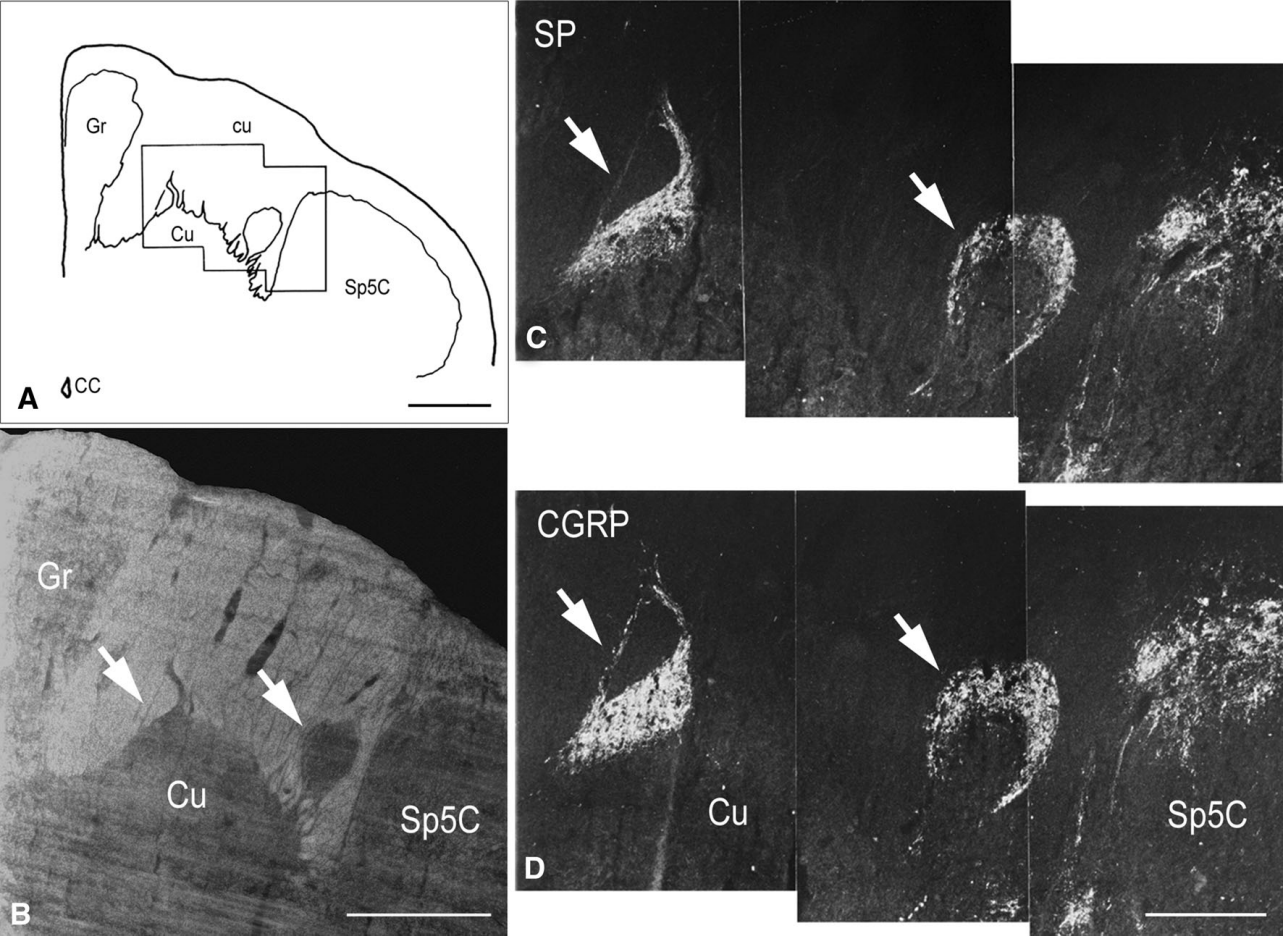
Full-term newborn, case 11. **A** Schematic drawing of the dorsal right quadrant of the caudal medulla oblongata transverse section shown in **B** and **C**; the *black frame* indicates the field shown in **C** and **D**. **B** Bright field orientation image of the territory of the cuneate nucleus (Cu) in a section immunostained for SP (detail in **C**). **C**, **D** Two adjacent sections immunostained for SP and CGRP, respectively, showing the dorsal part of the caudal Cu and the dorsomedial part of the spinal trigeminal nucleus, caudal part (Sp5C). *Arrows* in **B**–**D** indicate the immunoreactive Cu regions. *CC* central canal, *cu* cuneate fascicle, *Gr* gracile nucleus. *Scale bars* **A**, **B** = 1 mm, **C** = **D** = 500 μm

**Fig. 3:**
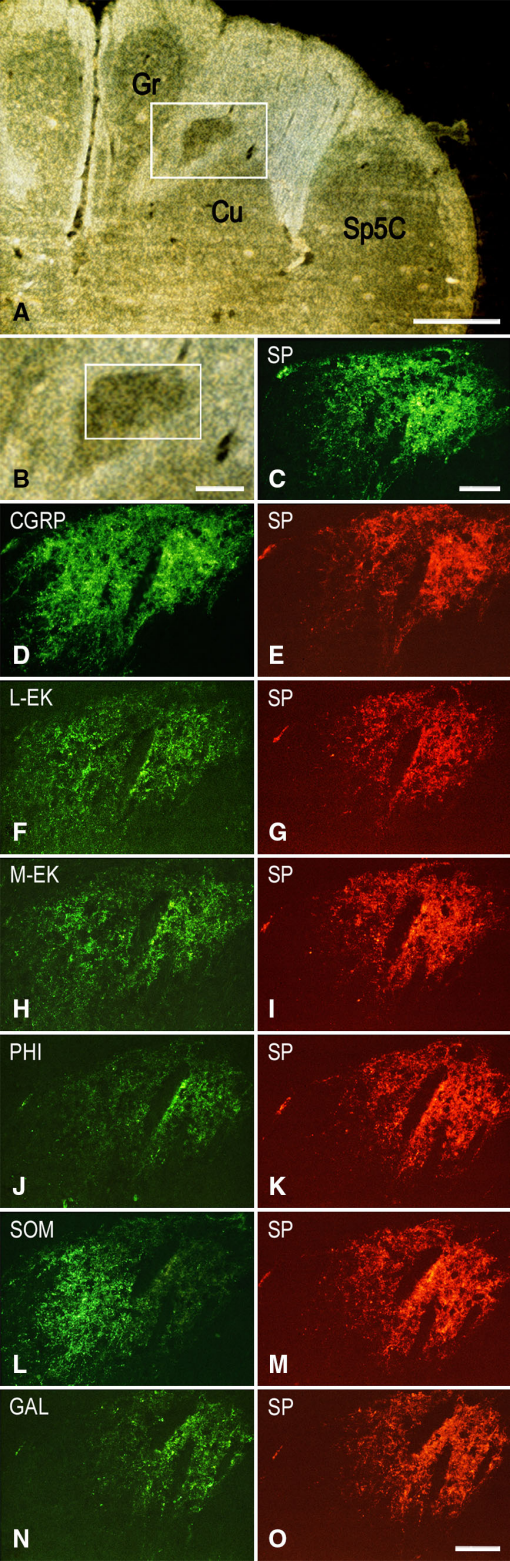
Full-term newborn, case 8. Series of seven consecutive sections of caudal medulla oblongata immunostained for SP (**A**, **B**, **C**) and double immunostained for CGRP (**D**), L-EK (**F**), M-EK (**H**), PHI (**J**), SOM (**L**), GAL (**H**) (DTAF fluorochrome), and SP (**E**, **G**, **I**, **K**, **M**, **O**, respectively) (TRITC fluorochrome). Images **A**, **B** and **C** belong to the same section immunostained for SP. **A** Bright field orientation image of the right dorsal quadrant: *white box* delineates the area shown in **B** at higher magnification. **B** Region of gray matter dorsomedial to the main cuneate nucleus (Cu); *white box* delineates the area shown in **C**. **C** *Dark field* image of the SP immunoreactivity. **D**, **E** to **N**, **O** *Dark field* paired micrographs of the area shown in **C** in six consecutive double immunostained sections. *Gr* gracile nucleus, *Sp5C* spinal trigeminal nucleus, caudal part. *Scale bars* **A** = 1 mm,** B** = 250 μm, **C** = 100 μm, **D**–**N** = **O** = 100 μm

**Fig. 4:**
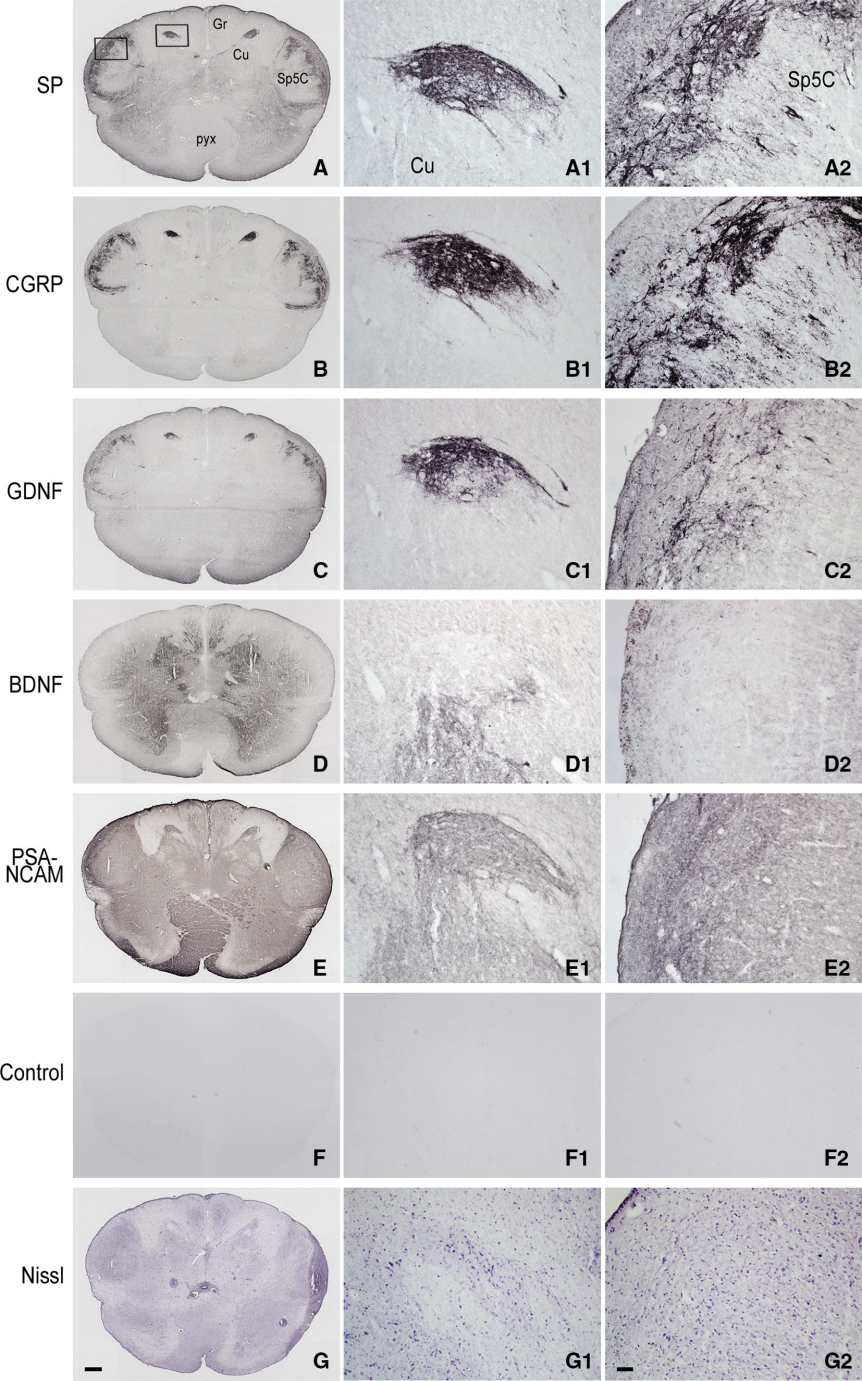
Pre-term newborn, case 5. **A**–**G** Series of seven consecutive sections of caudal medulla oblongata immunostained for SP (**A**), CGRP (**B**), GDNF (**C**), BDNF (**D**), PSA-NCAM (**E**) and as control (preabsorbed anti-PSA-NCAM primary antibody; preabsorbed antibodies against the other molecules yielded a similar outcome), and Nissl stained with cresyl violet (**G**). *Boxes* in **A** delineate the areas shown in **A**_**1**_–**G**_**1**_ and **A**_**2**_–**G**_**2**_. **A**_**1**_–**G**_**1**_ Higher magnification of the dorsal gray matter area in the left side cuneate nucleus (Cu) detectable in **A**–**G** sections, respectively. **A**_**2**_–**G**_**2**_ Detail at higher magnification of the left side substantia gelatinosa of the spinal trigeminal nucleus, caudal part (Sp5C), detectable in **A**–**G** sections, respectively. *Gr* gracile nucleus, *pyx* pyramidal decussation. *Scale bars* **A**–**F** = **G** = 500 μm, **A**_**1**_–**G**_**1**_ and **A**_**2**_–**F**_**2**_ = **G**_**2**_ = 50 μm

**Fig. 5:**
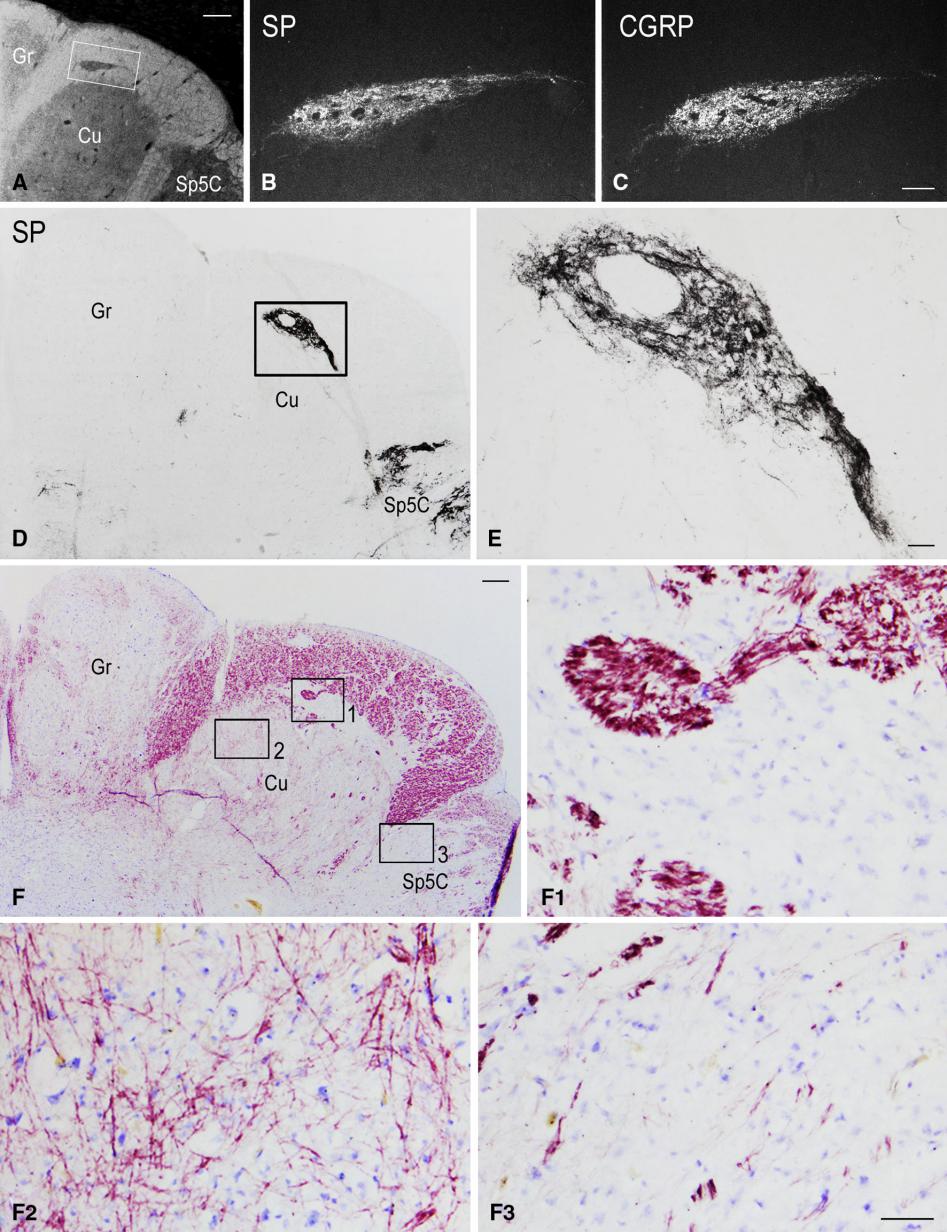
Full-term newborn, case 11 (**A**–**C**) and 9 (**D**–**F**_**3**_). **A** Bright field orientation image of the territory of the right cuneate nucleus (Cu) in a section of caudal medulla oblongata immunostained for SP (detail in *darkfield* in **B**): *white box* indicates area shown at higher magnification in **B**. **B**, **C** Two adjacent sections immunostained for SP and CGRP, respectively, showing the immunoreactive region of gray matter dorsal to the main Cu. **D** Right dorsal quadrant of a section immunostained for SP; the strongly immunoreactive area present along the dorsal border of the Cu (*box*) is shown at higher magnification in **E**. **F** Right dorsal quadrant of a section consecutive to **D** stained for myelin and Nissl; *boxes 1, 2* and *3* outline fields of the SP-immunoreactive Cu subregion, main Cu and caudal spinal trigeminal nucleus (Sp5C) substantia gelatinosa, respectively, shown at higher magnification in **F**_**1**_– **F**_**3**_. *Gr* gracile nucleus. *Scale bars* **A** = 500 μm, **B** = **C** = 100 μm, **D** = **F** = 250 μm, **E** = 50 μm, **F**_**1**_, **F**_**2**_ = **F**_**3**_ = 250 μm

**Fig. 6:**
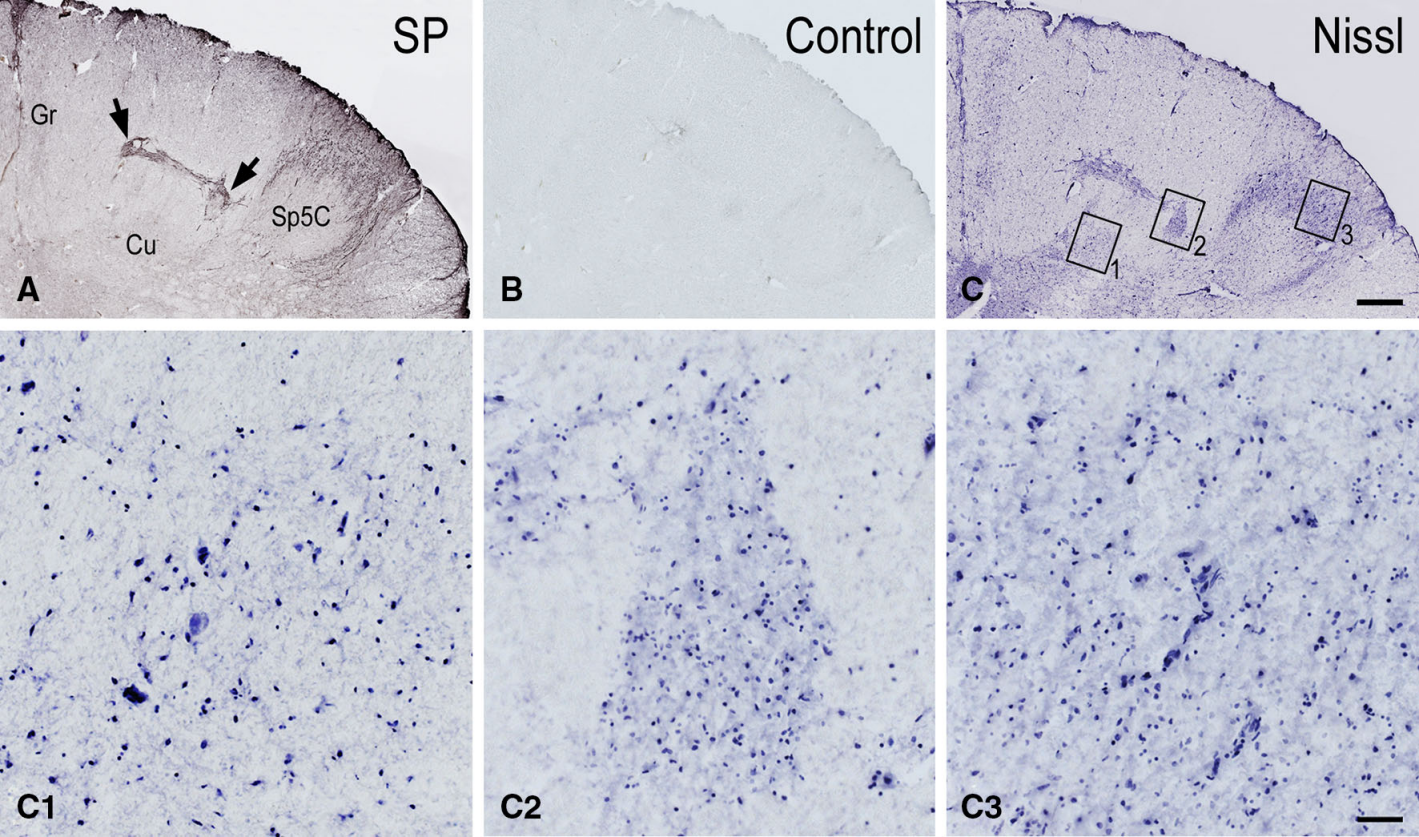
Adult, case 18. **A**–**C** Right dorsal quadrant of three consecutive sections of caudal medulla oblongata immunostained for SP (**A**) and as control (preabsorbed anti-SP primary antibody) (**B**), and Nissl stained (**C**). Details at higher magnifications of the Nissl stained main cuneate nucleus (Cu) (*box 1* in **C**), a region of the Cu territory immunoreactive to SP (*box 2* in **C**, compare to **A**), and the caudal spinal trigeminal nucleus (Sp5C) substantia gelatinosa (*box 3* in **C**) are shown in **C**_**1**_– **C**_**3**_, respectively. *Arrows* in **A** point to two SP-immunostained regions connected by a bridge of immunoreactive gray matter. *Gr* gracile nucleus. *Scale bars* **A**, **B** = **C** = 500 μm; **C**_**1**_, **C**_**2**_ = **C**_**3**_ = 50 μm

**Fig. 7:**
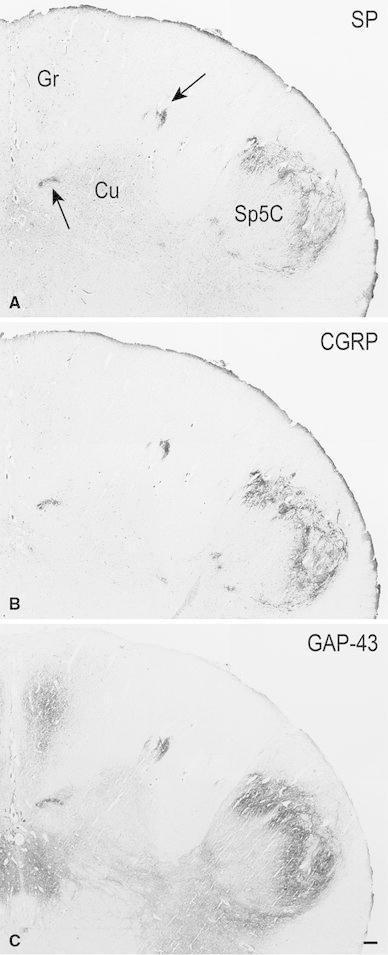
Adult, case 19. **A**–**C** Right dorsal quadrant of three consecutive sections of caudal medulla oblongata immunostained for SP (**A**), CGRP (**B**) and GAP-43 (**C**). Immunoreactvity for the three molecules occurs in two regions of the cuneate nucleus (Cu) (pointed out by *arrows* in **A**), as in the superficial layers of the spinal trigeminal nucleus, caudal part (Sp5C). *Gr* gracile nucleus. *Scale bar* **A**, **B** = **C** = 250 μm

**Fig. 8:**
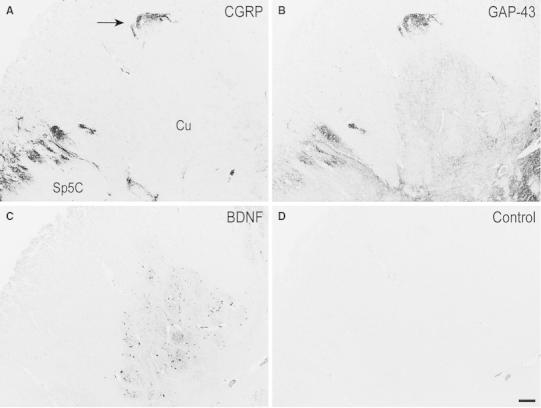
Adult, case 14. **A–D** Left side cuneate nucleus (Cu) and dorsomedial part of the caudal spinal trigeminal nucleus (Sp5C) substantia gelatinosa in four consecutive sections of caudal medulla oblongata immunostained for CGRP (**A**), GAP-43 (**B**), BDNF (**C**), and as Control (preabsorbed anti-BDNF primary antibody; preabsorbed antibodies against the other molecules yielded a similar outcome). Note how the immunoreactivity of the dorsal region of the Cu (*arrow* in **A**) goes in parallel to that of the substantia gelatinosa of the Sp5C for the three molecules. *Scale bar* **A**–**C** = **D** = 250 μm

**Fig. 9:**
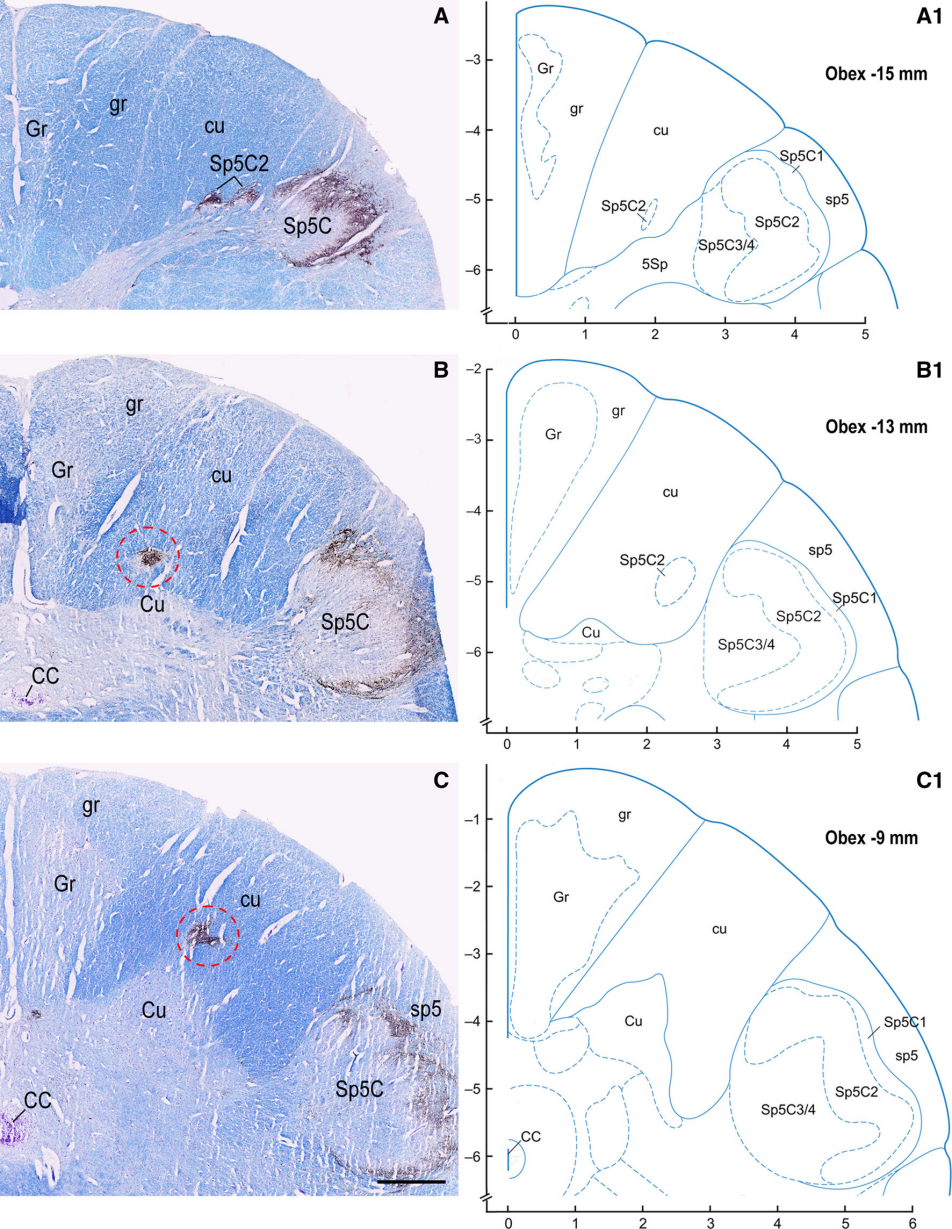

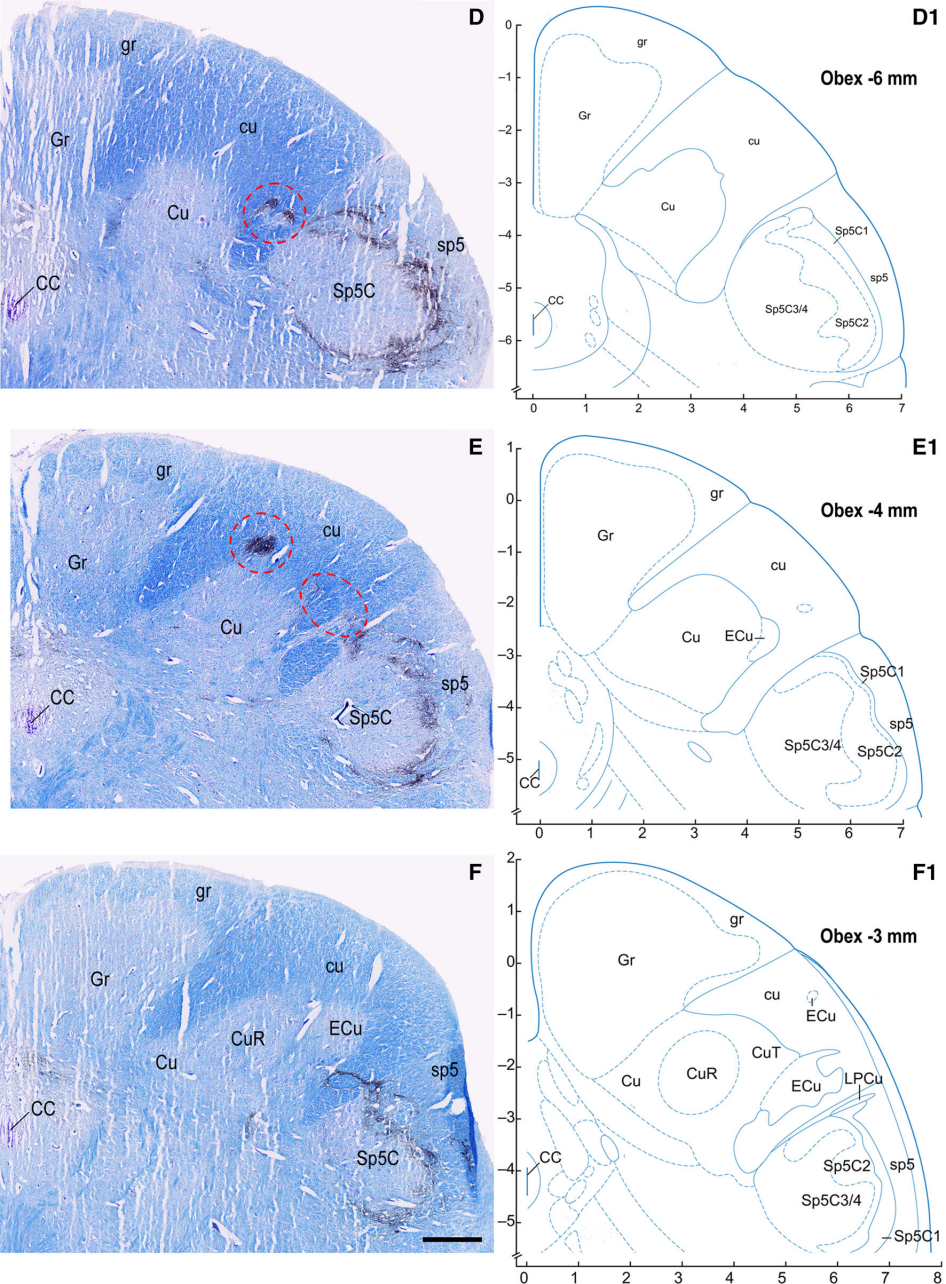
Adult, case 15. **A**–**F** Right dorsal quadrant of caudal medulla oblongata sections immunostained for CGRP and counterstained with Kluver-Barrera. **A**_**1**_–**F**_**1**_ diagrams modified from Paxinos et al. (2012) at levels indicated as distance from obex, matching those shown in **A**–**F**. Gray matter regions endowed with CGRP immunoreactivity and located medially (**B**), dorsally (**C**, **E**) and laterally (**D**, **E**) in the cuneate fascicle (cu) near the cuneate nucleus (Cu) border are encircled in red. Note that similar regions do not appear in the matching level diagrams. *CC* central canal; *CuR* cuneate nucleus, pars rotunda; *CuT* cuneate nucleus, pars triangularis; *ECu* external cuneate nucleus; *Gr* gracile nucleus; *gr* gracile fasciculus; *sp5* spinal trigeminal tract; *Sp5C* spinal trigeminal nucleus, caudal part; *Sp5C1* spinal trigeminal nucleus, caudal part, lamina 1; *Sp5C2* spinal trigeminal nucleus, caudal part, lamina 2; *Sp5C3/4* spinal trigeminal nucleus, caudal part, lamina 3/4. *Scale bar* **A**, **B** = **C** = 1 mm; **D**, **E** = **F** = 1 mm

In the caudal human medulla oblongata of all examined specimens, from fetal and young postnatal age (Figs. 1, 2, 3, 4, 5) to adult life (Figs. 6, 7, 8, 9), at levels between the pyramidal decussation and the obex, the territory of the cuneate fascicle and nucleus contains distinct areas of gray matter that differ from the remaining dorsal column nuclear complex with respect to their immunoreactivity to the examined substances. Networks of varicose filaments and dot-like structures, interpreted as nerve fibers and terminals, are revealed in them by immunostaining for the neuropeptides, GDNF, PSA-NCAM, and GAP-43; in addition, immunoreactivity to PSA-NCAM also shows profiles of cell bodies and proximal processes, suggestive of perikarya membrane labeling. Nerve fibers and terminals compose plexuses of variable density for the different markers, from very thick (SP, CGRP) to rather dense plexuses (SOM, M- and L-EK, GDNF, PSA-NCAM, GAP-43), or networks of moderate density (GAL, PHI). In these areas, no immunolabeling occurs for BDNF. By contrast, the remaining territory of the main Cu hosts scarce varicose fibers immunoreactive to the neuropeptides and GDNF, a thin mesh of filaments stained for GAP-43, rare filaments and profiles of cell bodies stained for PSA-NCAM, and strongly labeled neuronal perikarya, fibers and terminals for BDNF. The following description will focus on the localization pattern of the examined substances in the areas of strong SP immunoreactivity, whereas that in the main Cu will not be further described. No immunostaining was detectable in control preparations. However, as we can not completely exclude the possibility of non-specific adhesion of primary antibodies to unknown tissue components, the labeling should be correctly considered as “substance-like immunoreactivity”.

Compared to the outcome in newborn tissue, density of immunoreactive structures slightly lessens in adult specimens; immunoreactivity to GAP-43 and PSA-NCAM, while decreasing substantially in several gray and white matter areas, yet persisted in these regions with no major age changes. No gender differences were observed.

The position and shape of the Cu gray matter subregions that are strongly immunoreactive to SP, as detected in 37 consecutive sections encompassing about 4.5 mm caudo-rostral extent of a full-term newborn specimen, is illustrated in three-dimensional rendering in Fig. 1A–D. The largest extent of the SP-immunoreactive fields occurs below the level of the external cuneate nucleus caudal pole (indicated in Fig. 1B) and concentrates in two distinct caudo-rostral segments, separated by an interval of about 1 mm. At higher levels, where pars rotunda and pars triangularis of the main Cu are present, the immunoreactivity is localized to very small areas within the dorsomedial cuneate fascicle and at the boundary with the gracile territory (Fig. 1E 19–35a), where it has the aspect of thin bundles of fibers; it is undetectable at levels rostral to the obex. Alternate sections stained for myelin show the topographical localization of the immunoreactive subregions (Fig. 1E). Depending on the level considered, the immunoreactivity is placed in the superficial part of a somewhat larger gray matter area. Such a distribution may be clearly appreciated in Figs. 1E 2–2a and 16–16a, 2A–C, 3A–C. Otherwise, the two territories almost coincide (Fig. 5A, B). Also, they may appear as part of the dorsal contour of the Cu, more or less protruding dorsally (Figs. 2C, 4A, A_1_, 5D, E, 7A), or within islands of gray matter entirely embedded in the cuneate fascicle (Figs. 3C, 5B). Two distinct SP-positive zones may appear in the horizontal plane, one located medially and the other one laterally along the dorsal boundary of the Cu (Figs. 2C, 6A, 7A). Although their position may be close to the neighboring nuclei, topographically they are clearly related to the Cu rather than to the gracile and spinal trigeminal nuclei. The medially located region may show a triangular or oval profile, whereas the lateral one is round-shaped. In both areas, immunoreactivity to SP is located along the periphery and, in the lateral one, is contained in a crescent-shaped superficial layer (Figs. 2C, 6A, 7A). The thick plexus of nerve fibers and terminals is alike that present in the adjacent substantia gelatinosa of the Sp5C (Figs. 2C, 7A). At appropriate levels, it may be seen that shoots of immunoreactive varicose fibers from the white matter immediately dorsal to the Sp5C substantia gelatinosa join the laterally located area (Fig. 1E 2) and that the medial and lateral fields are connected by a bridge of gray matter that contains immunoreactive fibers (Fig. 6A, C). At some levels, a single area of immunostained gray matter occurs dorsomedial or dorsal to the main Cu; it is roughly triangular in shape and grows oval and transversely elongated at more rostral levels (Figs. 1E 14–16a, 3A–C, 4A, A_1_, 5A, B).

 sections shows that the Cu subregions with strong immunoreactivity to SP contain rare myelinated fibers (Fig. 5F, F_1_). In a similar way, the SP-immunoreactive substantia gelatinosa of the Sp5C (Fig. 5D) contains sparse stained fibers (Fig. 5F, F_3_), whereas numerous myelinated fibers can be seen running across the territory of the main Cu (Fig. 5F, F_2_). Nissl staining of adult tissue (Fig. 6) indicates that in the dorsal Cu subregions the cells may be smaller and more closely packed than in the main Cu (Fig. 6C–C_2_), their histological aspect appearing rather similar to that of the Sp5C substantia gelatinosa (Fig. 6C_2_, C_3_).

Together with SP, immunoreactivity to CGRP, L- and M-EK, PHI, SOM, GAL occurs in these regions with distinctive distribution and density for each of them (Figs. 2, 3, 4A, A_1_, B, B_1_, 5A–C, 7A, B, 8B). Double immunostaining for each peptide and SP in a series of adjacent sections (Fig. 3D–O) allows comparison of the localization pattern of neuropeptides with respect to one another. Structures immunoreactive for CGRP overlap those positive for SP, but are more abundant and reach more deeply into the area (Fig. 3D, E); immunoreactivity for L- and M-EK has a distribution similar to that for CGRP, but density of EK-positive elements is obviously lower compared to CGRP- (Fig. 3, compare F, H with D) and SP-positive ones (Fig. 3F–I); staining for PHI is distributed as that for SP, but is comparatively scarce (Fig. 3J, K); SOM-positive elements are located more deeply than those positive to SP and hardly overlap with them (Fig. 3L, M); immunoreactive material for GAL, though much less dense, overlaps that for SP (Fig. 3N, O). Figures 2, 4A–B_2_, 5, 7A, B show that the codistribution of SP and CGRP in the Cu subregions parallels that present in the Sp5C superficial layers, the immunostaining for CGRP being more intense than that for SP in both sites. With a similar strict correspondence in aspect, localization and density of immunoreactive structures detectable in the Sp5C superficial layers, the Cu subregions are also immunoreactive to GDNF (Fig. 4C–C_2_), PSA-NCAM (Fig. 4E–E_2_) and GAP-43 (Figs. 7C, 8B), and not to BDNF (Figs. 4D–D_2_, 8C). The aspect and position of the gray matter subregions in the adult medulla oblongata, as they appear in CGRP-immunostained and Kluver-Barrera counterstained tissue sections, are illustrated in Fig. 9 at some representative levels in caudo-rostral sequence. As reference, matching level diagrams, modified from Paxinos et al. (2012), are drawn aside. At caudalmost levels (Fig. 9A), below the Cu caudal pole, two immunoreactive small areas occur medial to the dorsomedial Sp5C substantia gelatinosa and immediately ventral to the cuneate fascicle. They have a different rostrocaudal extent, and only one of them or both, as in Fig. 9A, may appear in the same section. Moving rostrally, a single small area appears, more medially located and entirely embedded within the ventromedial cuneate fascicle; it enlarges rostrad and the immunoreactivity occupies its dorsolateral part (Fig. 9B, C). This area, both for its position and localization of immunoreactive material, is very similar to the one shown in newborn tissue in Figs. 3 and 4. Further rostrally and for at least 2 mm, it is not present (Fig. 9D). Laterally, a round-shaped region appears, which contains the immunoreactive elements in a semilunar superficial part. This region is very similar to that detected in newborn tissue and shown in Fig. 2. Rostrad, it grows larger and seems to split in two portions (Fig. 9D), which, at a higher level, diverge further, the medial one approching the dorsal border of the Cu (Fig. 9E). At the same point, a gray matter immunoreactive area appears again dorsal to the main Cu (Fig. 9E), roughly aligned with the one present at lower levels. It does not extend much rostrally and is not present at Cu middle level, when pars rotunda and pars triangularis are visible (Fig. 9F). Shoots of immunoreactive fibers bridge the ventrolateral cuneate fascicle, connecting the dorsalmost territory of the trigeminal spinal tract to the lateral border of Cu (Fig. 9F). At this and higher levels up to the obex, the gray matter immunoreactivity dies down, whereas thin fiber bundles appear in the dorsomedial cuneate fascicle, as shown in the newborn tissue in Fig. 1E 30, 35. Alternate sections immunostained for SP and counterstained with cresyl violet show a similar distribution for the peptide (not shown).

## Discussion

The data presented confirm our previous reports on the occurrence of gray matter areas that contain a rich plexiform network of intense SP immunoreactivity in the territory of the human Cu and cuneate fascicle, located at levels spanning from the pyramidal decussation to the obex and detectable throughout life, from fetal to adult age (Del Fiacco et al. 1983, 1984). These areas begin to appear at the level of the caudal reticular pole of the main Cu and continue rostrally, placed along the dorsal border of the main Cu. At their larger extent, they appear located at two separated caudo-rostral levels, both below the level of the external Cu caudal pole. At middle level of the main Cu, when pars rotunda and pars triangularis are present, they grow small and the immunoreactivity is restricted to spot-like regions embedded in the dorsomedial cuneate fascicle. Detailed descriptions of the human dorsal column nuclei in Nissl and acetylcholinesterase (AChE) stained transverse sections have been provided in recent years (Paxinos et al. 1990; Paxinos and Huang 1995; Koutcherov et al. 2004). The possibility that some intensely AChE reactive regions within the territory of the Cu and cuneate fascicle correspond to those we described earlier as strongly immunoreactive to SP has been put forward (Paxinos et al. 1990, 2012; Koutcherov et al. 2004). Comparison of the present data with the most recent diagrammatic representation of the human brainstem by Paxinos et al. (2012) suggests that the islands of gray matter indicated by those authors as trigeminal substantia gelatinosa (abbreviated as Sp5C2) within the ventral cuneate fascicle may correspond to the small immunoreactive regions occurring medial to the dorsomedial end of the Sp5C substantia gelatinosa at levels immediately caudal to the Cu caudal pole. By contrast, the immunoreactive area that starts to appear at the Cu caudal pole is definitely more medially situated than the regions seen below and the Sp5C2 drawn in diagrams. Moreover, CGRP immunoreactivity occupies only its dorsolateral part, suggesting that the whole area may correspond to a territory wider than a mere substantia gelatinosa. Further rostrally, the immunoreactive gray matter subregions present in the caudal Cu are not identified and drawn as specific territories in those diagrams. We detected clusters of few large cells well stained with Nissl in the cuneate fascicle, which correspond well to the very small islands of external Cu drawn by Paxinos et al. (2012). However, contrary to the speculation by Paxinos et al. (2012) that they may correspond to the SP-positive regions we described earlier, their neuropil is not immunoreactive to SP or CGRP and, conversely, the regions identified by their immunoreactivity to the examined substances host small neurons.

Thus, the regions described here appear distinct from the known subdivisions of the dorsal column nuclear complex present at their same level, including the medial pericuneate nucleus, which is ventral to the dorsal column nuclei, and the lateral pericuneate nucleus, which is ventral and lateral to the external Cu (Paxinos et al. 1990, 2012). Even more obviously, they are distinct from the nuclei of the complex located above the obex level, such as the paratrigeminal nucleus and nucleus X. The present work further shows that immunoreactivity to neuropeptides CGRP, L- and M-EK, GAL, SOM and PHI, plasticity proteins PSA-NCAM and GAP-43, and trophin GDNF, but not BDNF, also occurs in these regions and that the aspect, distribution pattern and density of immunostained elements for all these molecules match up strictly with those detected in the Sp5C. Such a strict resemblance may be further appreciated by comparing the present data with our previous descriptions of the Sp5C immunoreactivity to the examined neuropeptides (Del Fiacco and Quartu 1994; Quartu et al. 1992; Quartu and Del Fiacco 1994), GAP-43 (Del Fiacco et al. 1994), GDNF (Del Fiacco et al. 2002) and PSA-NCAM (Quartu et al. 2008). Nissl and myelin stainings support the concept of a similar histological organization between the detected immunoreactive fields and the Sp5C substantia gelatinosa. We consider that the present observations provide a clear-cut indication for the existence of distinct subregions (which may possibly be considered as a single whole structure) in the human dorsal column nuclear complex, placed in the territory of the main Cu and cuneate fascicle, never formally determined so far and whose neurochemical anatomy bears a striking resemblance to that of the spinal and brainstem sensory nuclei committed to transmit protopathic stimuli, including pain. At various levels, they may appear composed of a deep part, almost devoid of immunoreactivity, and a superficial part where the immunoreactivity for the examined substances parallels that present in the superficial layers of the Sp5C (present data; Del Fiacco et al. 1984; Chigr et al. 1989; Rikard-Bell et al. 1990; Unger and Lange 1991; Quartu et al. 1992, 1999, 2008; Del Fiacco and Quartu 1994; Quartu and Del Fiacco 1994; Del Fiacco et al. 1994; 2002; Yan et al. 1997; Kawamoto et al. 2000) and spinal dorsal horn (Cuello et al. 1976; De Lanerolle and LaMotte 1982; Anand et al. 1984; Chung et al. 1989; Unger and Lange 1991; Nacimiento et al. 1993; Bonfanti et al. 1992; Jongen et al. 1999; Kawamoto et al. 2000; Del Fiacco et al. 2002; Schoenen and Faull 2004). Thus, the observed immunolocalizations suggest to explore the possibility that these subregions contain a superficial laminar pattern in their structural organization.

Vast amount of literature on the chemical anatomy of the somatosensory system in different animal species, including man, shows how immunoreactivity to the examined molecules, abundant in the superficial laminae of the spinal dorsal horn, Sp5C, and solitary nucleus, is generally scarce and dispersed throughout in the dorsal column nuclei (see references in Introduction; Rustioni and Weinberg 1989; Broman 1994). Several experimental studies report a significant intensification of the immunoreactivity to some neuropeptides in dorsal column nuclei after peripheral nerve damage (Hoeflinger et al. 1993; Zhang et al. 1993; Noguchi et al. 1995; Ma and Bisby 1997; Miki et al. 1998; Yeh et al. 2008) and in aging (Kitagawa et al. 2005); moreover, according to the well-documented notion that GAP-43 synthesis and axonal transport raise in neurons responding to peripheral nerve injury (Skene 1984; Bisby 1988), increased levels of the protein were observed in monkey Cu after median nerve lesion and repair (Jain et al. 1995) and in rat gracile nucleus after sciatic nerve crush injury (Woolf et al. 1990). In this respect, it is to be underlined that our observations were made on normal specimens and that, as a rule, the immunoreactivity in specimens from aged subjects was less pronounced or less densely packed than in young tissue.

In agreement with their preferential localization to sensory nuclei that receive protopathic sensory signals, the examined neuropeptides (see above for references), GDNF (Holstege et al. 1998) and GAP-43 (Tetzlaff et al. 1989) are functionally related to the neurotransmission of protopathic sensibility, including pain, and are mostly contained there in unmyelinated or thinly myelinated primary afferent fibers and in local circuit neurons. Moreover, typically PSA-NCAM persists in adult tissue in the superficial laminae of the spinal dorsal horn (Bonfanti et al. 1992) and Sp5C (Quartu et al. 2008). Though, in the rat, BDNF may play a role as an anterograde trophic messenger in unmyelinated or thinly myelinated primary afferent fibers to spinal dorsal horn neurons (Michael et al. 1997), in our hands BDNF immunoreactivity is undetectable in the identified subregions of the human Cu as well as in the trigeminal substantia gelatinosa and the latter finding appears in agreement with what reported in the rat Sp5C by Yan et al. (1997).

By contrast, the possibility that some of the examined neuropeptides play a role in the sensory modalities traditionally ascribed to the dorsal column nuclei, namely fine somatic tactile and proprioceptive perception, may also be considered. In fact, CGRP-positive fibers have been shown to innervate the light touch mechanoreceptor Meissner’s corpuscles (Ishida-Yamamoto et al. 1988), and SP-positive fibers also occur in human (Dalsgaard et al. 1983; our unpublished observation) and rat Meissner’s corpuscles (Ishida-Yamamoto et al. 1988), and in other mechanoreceptors such as human Ruffini’s and Pacini’s corpuscles (Ide et al. 1996). Moreover, the possible involvement of SP in the neurotransmission of proprioceptive stimuli is suggested by its localization in the human nucleus dorsalis of Clarke (Pioro et al. 1984).

The dorsal column-medial lemniscus system has classically been viewed as a pathway devoted to transmission of fine touch, vibratory sense and proprioception, and the dorsal columns have been considered as composed mostly of thick myelinated primary sensory fibers. However, it is now well established that a substantial amount of the dorsal column fibers are thin and unmyelinated (Langford and Coggeshall 1981; Chung and Coggeshall 1985; Chung et al. 1987), their proportion exceeding 25 % in the human sacral spinal cord (Briner et al. 1988). In the rat, many of these fibers are of primary afferent origin and, among the latter, most are immunoreactive to CGRP (McNeill et al. 1988; Tamatani et al. 1989; Patterson et al. 1990) and a smaller number to SP (Tamatani et al. 1989). In human tissue, SP-immunoreactive fibers run longitudinally along the dorsal border of the cuneate fascicle and bend obliquely at its ventrolateral border, towards the adjacent Cu territory (Del Fiacco et al. 1984). These observations were confirmed in the present study; CGRP-positive fibers were also seen with similar position and orientation (not shown). The possibility of a primary sensory origin other than spinal ganglia is suggested by the presence of CGRP and SP-immunoreactive fibers extending from the territory of the dorsal spinal trigeminal tract to the ventrolateral cuneate fascicle and adjacent nucleus; such an observation is in agreement with the well-established notion of trigeminal primary afferent input to the Cu (see Murfert and Rajchert 1991 and references therein). In keeping with such a composition of the white matter is the fact that nociceptive responses have been recorded in the dorsal column nuclei (Ferrington et al. 1988; Cliffer et al. 1992; Usunoff et al. 2006; Zhao et al. 2011). Moreover, the dorsal column pathway appears to be involved in chronic neuropathic pain states (Ossipov et al. 2000). Of particular interest is the postsynaptic dorsal column (PSDC) pathway, a fiber system originating from spinal cord cells postsynaptic to primary afferent fibers (Rustioni 1976; Giesler et al. 1984; Cliffer and Willis 1994). The PSDC pathway has a major role in visceral nociception (Cliffer and Giesler 1989; Berkley and Hubscher 1995; Hirshberg et al. 1996; Feng et al. 1998; Al-Chaer et al. 1999; Willis et al. 1999; Wang et al. 1999) and its surgical disruption represents a successful strategy in the control of visceral cancer pain in patients (Hirshberg et al. 1996; Nauta et al. 1997; Kim and Kwon 2000; Gildenberg 2001). Experimental findings show that the PSDC pathway contains peptidergic fibers (Conti et al. 1990). Particularly in view of the possible clinical implications, namely the prospect of a pharmacological rather than surgical pain therapy (see Wang et al. 2011), it is important to define accurately the chemistry of this pathway. Based on their neurochemical features, it is tempting to speculate the involvement of the Cu subregions in the processing of pain stimuli and possibly in visceral nociception. On the other hand, comparative analyses in different species across mammalian Orders indicate that the PSDC pathway, rather than to discrete areas, has a general widespread distribution in the Cu (Cliffer and Giesler 1989; Cliffer and Willis 1994) and projects to both cuneate and gracile nuclei (Giesler et al. 1984; Palecek et al. 2003a, b). In particular, in the monkey, the PSDC pathway has been show to reach all parts of the Cu, including pars rotunda, and the external Cu (Cliffer and Willis 1994).

In conclusion, we show that immunoreactivity to a number of molecules with major roles in protopathic neuronal transmission, trophism and plasticity concentrates in restricted areas of the human Cu, mostly located at caudal levels of the nucleus, along its dorsal edge or embedded in the white matter of the cuneate fascicle, and propose that such subregions may represent a distinct subdivision of the human dorsal column nuclei with neurochemical and histological features analogous to those of the protopathic and nociceptive sensory nuclei. Their occurrence in the human Cu poses numerous questions that demand further investigations. For instance, as among the subdivisions of the dorsal column nuclear complex we could detect them exclusively within the territory of the Cu, whether or not their functional involvement is committed to serve both the upper and lower part of the body remains to be elucidated. Studies should be aimed at clarifying whether or not they are exclusively found in the human brain, since similar clear-cut subregions, endowed with the described neurochemical properties, have not been reported in other species. The possibility may be considered that they may have gone unnoticed. In fact, in their wider extent, they appear concentrated at two longitudinal segments, and a thorough inspection of the medulla oblongata is required to detect them. Crucial would be their identification in experimental animals, and particularly in primates, as studies aimed at clarifying their fine structure, chemistry, hodology and functional implications could then be envisaged.
